# Implementation of a Heart Failure Telemonitoring System in Home Care Nursing: Feasibility Study

**DOI:** 10.2196/11722

**Published:** 2019-07-26

**Authors:** Emily Seto, Plinio Pelegrini Morita, Jonathan Tomkun, Theresa M Lee, Heather Ross, Cheryl Reid-Haughian, Andrew Kaboff, Deb Mulholland, Joseph A Cafazzo

**Affiliations:** 1 Institute of Health Policy, Management and Evaluation University of Toronto Toronto, ON Canada; 2 eHealth Innovation University Health Network Toronto, ON Canada; 3 Techna Institute University Health Network Toronto, ON Canada; 4 School of Public Health and Health Systems University of Waterloo Waterloo, ON Canada; 5 Institute of Biomaterials and Biomedical Engineering University of Toronto Toronto, ON Canada; 6 Ted Rogers Centre for Heart Research Peter Munk Cardiology Centre Toronto, ON Canada; 7 Department of Medicine University of Toronto Toronto, ON Canada; 8 Professional Practice, Knowledge and Innovation ParaMed Home Health Care Toronto, ON Canada; 9 CellTrak Technologies, Inc Schaumburg, IL United States

**Keywords:** patient monitoring, home care services, heart failure, mobile phone, feasibility studies

## Abstract

**Background:**

Telemonitoring (TM) of heart failure (HF) patients in a clinic setting has been shown to be effective if properly implemented, but little is known about the feasibility and impact of implementing TM through a home care nursing agency.

**Objective:**

This study aimed to determine the feasibility of implementing a mobile phone–based TM system through a home care nursing agency and to explore the feasibility of conducting a future effectiveness trial.

**Methods:**

A feasibility study was conducted by recruiting, through community cardiologists and family physicians, 10 to 15 HF patients who would use the TM system for 4 months by taking daily measurements of weight and blood pressure and recording symptoms. Home care nurses responded to alerts generated by the TM system through either a phone call and/or a home visit. Patients and their clinicians were interviewed poststudy to determine their perceptions and experiences of using the TM system.

**Results:**

Only one community cardiologist was recruited who was willing to refer patients to this study, even after multiple attempts were made to recruit further physicians, including family physicians. The cardiologist referred only 6 patients over a 6-month period, and half of the patients dropped out of the study. The identified barriers to implementing the TM system in home care nursing were numerous and led to the small recruitment in patients and clinicians and large dropout rate. These barriers included challenges in nurses contacting patients and physicians, issues related to retention, and challenges related to integrating the TM system into a complex home care nursing workflow. However, some potential benefits of TM through a home care nursing agency were indicated, including improved patient education, providing nurses with a better understanding of the patient’s health status, and reductions in home visits.

**Conclusions:**

Lessons learned included the need to incentivize physicians, to ensure streamlined processes for recruitment and communication, to target appropriate patient populations, and to create a core clinical group. Barriers encountered in this feasibility trial should be considered to determine their applicability when deploying innovations into different service delivery models.

## Introduction

### Background

Heart failure (HF) is associated with poor health outcomes and high costs largely because of frequent hospitalizations [1-4]. Tools, such as telemonitoring (TM), have been proposed to improve clinical management and self-care of patients with HF. TM is the use of information technology to monitor patients at a distance (ie, at home) while empowering them to participate in their own care [5].

Recent systematic reviews have found that TM for HF management reduces mortality risk and hospital readmissions and more frequent transmission of patient data increases its effectiveness [6,7]. However, several studies, including 3 notable large-scale trials, have failed to confirm the benefits of TM [8-10]. This inconsistency in the findings of TM on HF outcomes can be attributed to the heterogeneity of the trials, including the characteristics of the intervention being studied, the characteristics of the patient population (eg, demographics and disease severity), and how the TM system is implemented.

Most previous large-scale trials of HF TM have been in the context of TM being embedded in specialty clinics (eg, HF clinics) or through primary care physicians’ offices [6,7]. However, an important supplemental health service for HF patients who are at high risk for hospitalization is home care nursing (ie, nurses who visit patients at their homes as required) because many are too unwell to travel [11]. Between scheduled home care nursing visits, patients often perform minimal or no self-care and can deteriorate quickly [11-14]. TM by home care nurses could provide a method to more closely monitor patients and increase the number of patients a particular nurse can manage. Preliminary studies indicate that TM by home care agencies can lead to improved outcomes [15,16]. It has also been found that TM through home care can be relatively equivalent to live home visits when it comes to managing HF [17]. However, the understanding of the potential feasibility of sustained HF TM embedded into a home care nursing agency’s services remains unclear [18,19].

### Objective

The objective of this research was to conduct a feasibility trial to investigate the feasibility and barriers associated with implementing a mobile phone–based TM system to monitor HF patients, led by general home care nurses through a home care nursing agency. The 2 main research questions for this study are as follows: (1) how feasible is it to integrate a mobile phone–based TM system into a home care nursing agency’s services? and (2) how feasible is it to conduct a future effectiveness trial of a mobile phone–based TM system within a home care nursing context?

This feasibility study was conducted in collaboration between a research and development center at a large university-affiliated hospital, a Canadian home care nursing agency, and a private company that provides an integrated care coordination platform used by the home care nursing agency. This integrated care coordination platform enables home care nurses to gain access to scheduling and patient information, as well as to document their home visits while in the field.

## Methods

### The Telemonitoring System

**Figure 1:**
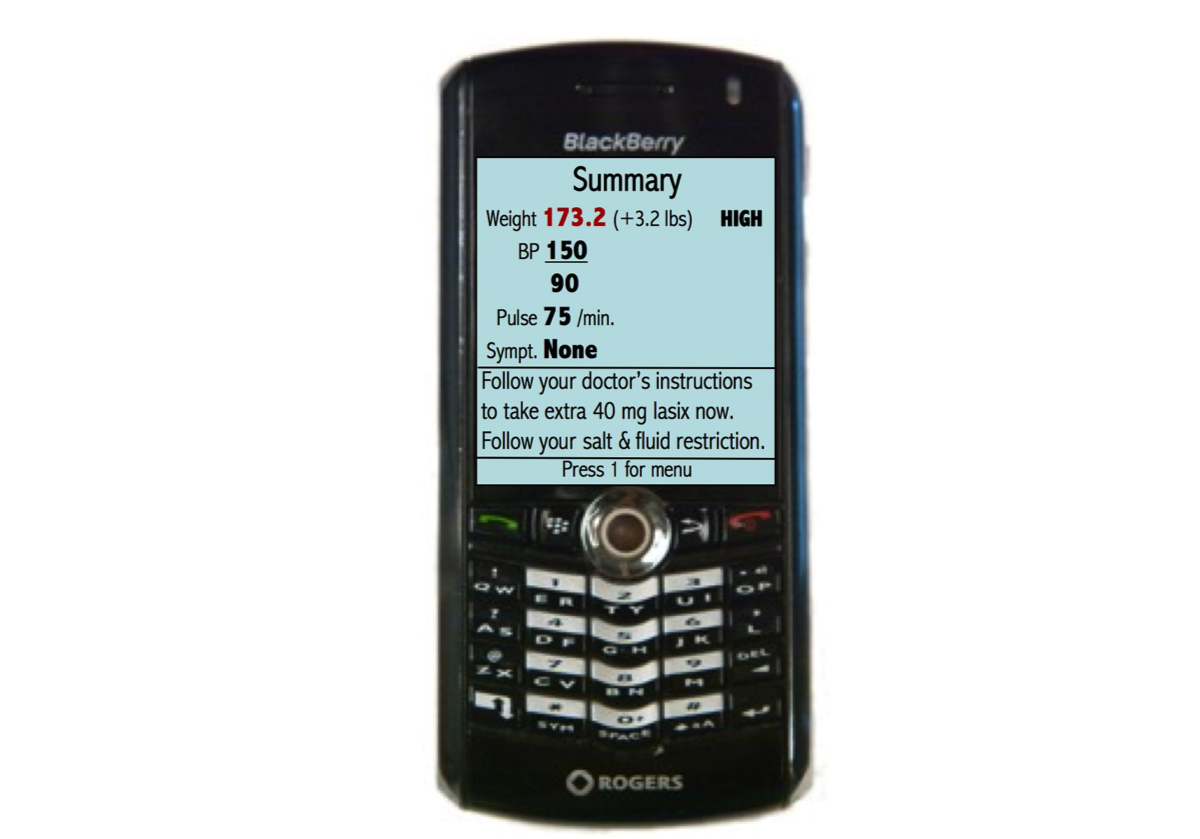
Screenshot of the TM mobile phone app. TM: telemonitoring.

Through a mobile phone app (see Figure 1), the TM system allows HF patients to monitor their health by recording weight and blood pressure measurements daily with Bluetooth-enabled home medical devices. The measurements are automatically and wirelessly transmitted to the mobile phone and then to a secure data server. Patients are asked to answer simple yes or no symptom questions on the mobile phone, such as whether they have more chest pain than usual or if they have more difficulty breathing at night than usual. Automated self-care instructions and advice are sent immediately to the patient based on their measurements and reported symptoms.

The TM system was combined with the integrated care coordination platform that was already being used by the home care agency. If the TM system detected signs of an exacerbation, an alert with all relevant data was sent to the home care agency through the integrated care coordination platform, where the alert was viewed by a portal administrator on an administrator dashboard. The alert was then assigned by an assignment coordinator to a specific home care nurse, and the alert information was forwarded by the portal administrator to the appropriate nurse’s mobile phone through the software that is part of the integrated care coordination platform. The nurse and patient’s physician were able to access all the patient’s TM data through a clinical dashboard through a secure website.

The alert threshold values can also be set and modified through the clinical dashboard. Physicians would determine the appropriate threshold values and can change the values themselves, or a home care nurse can change the values once they are confirmed by the responsible physician.

### Patient and Physician Recruitment

The feasibility study was conducted through a specific office of the home care agency because of it having the highest number of HF patients. The original intent was to partner with the local community care access center to funnel eligible patients into the trial. Unfortunately, the community care access center was not able to participate in the study because of commitments with other research studies. Therefore, the plan was changed to recruit 10 to 15 HF patients through physicians who would identify eligible patients. The patients would be enrolled into the home care services as private patients (nurses would need to be paid through research study funds). The intent was to identify physician participants through the hospital cardiologists and by managers at the home care agency. The managers contacted local family physicians and faxed introduction letters regarding the study. In addition, a letter from a hospital cardiologist was sent to several local cardiologists asking them to participate. Approval from the hospital research ethics board was obtained before commencement of the study (REB 12-0525-AE).

### Patient Participant Eligibility Criteria

To be eligible for the study, patients had to be English speaking; diagnosed with congestive HF of New York Heart Association (NYHA) Class II or higher; aged older than 18 years; not already on home care services; residing in the study region of Oshawa, Ontario; and able to perform self-measurement tasks (eg, stand on the provided weight scale). Patients deemed unable to comply with the TM program (eg, because of vision problems or decreased cognitive function such as advanced memory loss) or unable to provide written informed consent were excluded from the study.

### Preparation for the Use of the Telemonitoring System

The proper integration and acceptance of the TM system into a home care nursing ecosystem required understanding the current workflow and training of multiple members of the home care team. The current workflow of the home care agency was mapped through discussion with relevant stakeholders and observation. Workflow maps were then developed that integrated the use of the TM system.

In terms of training, it was determined that all home care agency staff would be trained to prevent lapses in coverage, especially during the night and weekend off-hours. The staff required to attend training consisted of portal administrators, assignment coordinators, nursing supervisors, off-hours supervisors, and nurses (over 50 individuals). A unified training presentation was developed to ensure that all staff knew each other's roles and the scope of the study. Comprehensive training packages were prepared, specific to each of the 3 key study roles (portal administrator, assignment coordinator, and nurse). The sessions took the form of a PowerPoint presentation followed by a period for questions and lasted approximately 1 hour. A recorded training video, frequently asked questions document, and training slides were posted on the internet, were password protected, and were shared with any staff members who could not attend a training session.

### The Telemonitoring System Protocol

During the first home visit (initial assessment) by the home care nurse, the nurse provided a training session on how to use the TM equipment, which included a mobile phone with the TM app loaded on it, a weight scale, and a blood pressure monitor. The app was designed to be intuitive to use by using a user-centered design process including end-user usability testing. However, if participants had difficulties using the TM equipment after the training session, they were encouraged to call the provided technical support phone number.

The initial and follow-up assessments were standard assessments performed by home care nurses and included questions asking about functional status and symptoms, which were part of the Health Outcomes for Better Information and Care (HOBIC) measures, which is an assessment collected to capture standardized client outcomes data related to nursing care in 4 sectors: acute care, long-term care, complex continuing care, and home care [20]. According to the standard of care, after the initial assessment, nurses visited the patients approximately once every 2 weeks (the same nurse followed a particular patient except if the nurse was unavailable). In addition, assessments through the telephone and additional home visits were conducted if deemed necessary by the nurses because of TM alerts. Telephone assessments were outside the standard of care and introduced as part of the intervention. Before a telephone assessment or additional home visit, approval was first obtained by the nurse supervisor (standard practice to obtain approval for additional home visits). After 3 to 4 months of TM, the participants were asked to mail back the TM equipment and were discharged from home care services. A home care nurse conducted a discharge assessment on each patient participant that included the HOBIC measures.

### Data Collection and Analysis

All patient participants and home care nurses involved in the study were interviewed after obtaining informed consent to gain their perspectives on the feasibility of integrating a mobile phone–based TM into home care nursing services (research question 1). Specifically, the study coordinator conducted short prestudy semistructured interviews that were approximately 10 min in duration with the patient participants to gather insight on their current clinical care and self-care. Patient participants and the home care nurses were also interviewed poststudy by the study coordinator to determine their experiences with the TM system and the perceived impact of the TM system on HF management. The poststudy semistructured interviews sought to evaluate the experience with the TM system based on concepts in the technology acceptance model (TAM) [21], namely to determine the external variables, perceived ease of use, perceived usefulness, attitude toward using the technology, and behavioral intention to use. The poststudy interviews lasted approximately 30 min in duration. The interview data and transcripts were transcribed and coded for emerging themes by TL and PM. The research team (ES, PM, TL, and JT) reviewed and discussed the emerging themes until consensus was reached.

Beyond the interviews, data on patient adherence of using the TM system from the TM system database were also collected to help answer research question 1. Finally, data from forms that the home care nurses filled out after each home visit and telephone call to the patient were collected and analyzed. These forms sought information regarding if the visit was a telephone encounter or a home nursing visit; if the nurses perceived the home visit to be necessary; if they thought the home visit could have been replaced by a telephone visit using the data from the TM system; if the TM data were useful during the home visit; if they perceived the data to be helpful; if the nurses thought the data helped eliminate the need for a home nursing visit; and any additional remarks (open ended) about the clinical encounter, alerts from the TM system, or other issues noted.

To investigate the feasibility of conducting a future effectiveness trial (research question 2), data from several other sources required for an effectiveness trial were collected. Patient participants were provided pre- and poststudy questionnaires that included the Self-Care of Heart Failure Index (SCHFI) [22-24] and the Minnesota Living with Heart Failure Questionnaire (MLHFQ) [25-27]. The SCHFI is a validated self-reported tool to measure self-care of HF patients using 15 items rated on a 4-point response scale, and the MLHFQ is a validated self-reported tool to measure the quality of life of HF patients using 21 items on a 6-point Likert scale. Pre- and poststudy HOBIC measures were also collected. The pre- and poststudy values for the SCHFI, MLFHQ, and HOBIC measures were compared using descriptive statistics. In addition, a historical chart review of the home care patients was conducted to collect data on hospital visits, reasons for home care visits, and any clinical remarks that were noted about the patient.

## Results

### Physician and Patient Recruitment

A single local cardiologist agreed to participate in the study despite extensive efforts, outlined above, to recruit other physicians. The cardiologist referred 6 patients with HF NYHA class II or III to the study; all 6 patients provided written consent to participate in the feasibility study. The cardiologist did not believe she had additional patients who were suitable for the study during the study period. Additional efforts were made to recruit further patients, which included contacting health care providers (family health team clinics, walk-in clinics, family doctor offices, and pharmacies) in an expanded catchment area through fax, letters, and follow-up phone calls. Furthermore, efforts were made to recruit patients directly through posted advertisements at community care centers and clinics. However, none of these techniques led to additional patient participants. The demographics of the patient participants, early experiences of TM, and the length of their study enrollment are presented in Table 1.

**Table 1:** Patient demographics and early negative experiences related to days enrolled.

Age (years)	Gender	Employment status	Size of household	Early negative experiences with TM^a^	Days enrolled
86	Male	Retired or not working	≤2 family members	No	Completed study (119 days)
57	Male	Retired or not working (returned to work during study period)	≥3 family members	No	Completed study (116 days)
72	Female	Retired or not working	≤2 family members	No	Completed study (125 days)
46	Male	Working full time	≥3 family members	Yes	Dropped out after 3 days
35	Male	Working full time	≥3 family members	Yes	Dropped out after 34 days
58	Female	Retired or not working	≥3 family members	Yes	Dropped out after 44 days

^a^TM: telemonitoring.

### Use of the Telemonitoring System

Of 6 patients, 3 dropped out of the study. Reasons cited by patients for dropping out included incompatibility of scheduling home care visits with working full time, feeling overwhelmed with repeated phone calls from nurses because of alerts, perceived intrusiveness of home visits by nurses, and patient feeling too physically weak to comply with taking daily morning measurements after being discharged from a hospital stay.

On average, patients adhered to taking their daily weight, blood pressure, and symptom measurements 72% of the days (only including days before dropping out of the study). The nurses performed a total of 31 scheduled home visits for all 6 patients. They reported that, in their judgment, 16 of the 31 home visits (52%) could have been replaced by phone call assessments supported by the TM data. The nurses reported that they used the TM data before the visit in 15 of the home visits (48%), used the data during the home visit in 17 of the home visits (55%), and did not use the data for 3 home visits (10%). In general, the nurses reported that the TM data were useful in 28 of the home visits (74%) for assessing the patient or providing informed care.

A total of 208 alerts were triggered throughout the study. There was a total of 5 alerts that were classified as *critical*, advising patients to call 9-1-1 or go to the emergency department. The nurses made a total of 38 phone calls to the patients because of alerts that were triggered by the TM system. The nurses reported that in 28 of the 38 phone calls (74%), the TM data were useful in assessing the patient over the phone. No home visits were made as a result of the triggered alerts.

A review of the nurses’ visit logs and final interviews found that nurses were able to speak to patients to verify their medication and symptoms over the telephone calls. During home visits, nurses were able to provide instructions to patients on proper blood pressure management techniques and engaged with patients regarding their diet, such as reducing sodium to decrease shortness of breath or using compression stockings to reduce pedal edema.

### Perceptions of the Telemonitoring System

All 6 patient participants were interviewed before using the TM system about their experience with HF. At the end of the study, semistructured interviews were conducted with the 3 patient participants who completed the study about their experiences with home care and perceptions of the TM system. The 2 home care nurses who were the most involved in the study and who regularly managed the patient participants were also interviewed about their experiences with the system to gain insights into the feasibility of implementing the HF TM system into their workflow and services.

### Perceptions by Nurses

#### Workflow Barriers

Both the interviewed nurses described issues associated with the TM program that related to workflow barriers of implementing TM. For example, certain patients were triggering alerts regularly because of inappropriate thresholds. Therefore, the nurses needed to contact the responsible physician to take the appropriate action or change the alert thresholds. However, the specialist physician was not always in their office and could not be reached by phone, and it was challenging to get a response to make the necessary changes in adjusting the TM system. One nurse stated:

The other thing I found difficult was getting a hold of the doctor. If you wanted to do things—it was difficult. We are always getting in touch with doctors from the community—it shouldn’t be as difficult as it was to get a hold of this one. We can get a hold of doctors and get a response the next day, even with surgeons. But with this doctor sometimes it took two days, which to me, if someone is having and showing signs of getting in distress, two days is too long. We can see the ship sinking but we can’t do anything about it if we can’t get a hold of the doctor.

One nurse described feeling frustrated in not being able to connect to the patient they needed to speak with:

I got an alert on him every day. He also had a cell phone...and never, ever picked up...I just left messages with what the issue was and explaining it.

Another issue was slow access to the patient information and alerts:

I would say about 40% of the time, it was a pain to try and get into the system...once you get an alert, you want an instantaneous alert, sometimes it would take longer than we would have liked.

In this excerpt, the nurse was referring to the technological aspect of the system and accessing the Web portal where the user can view patients’ vitals on their mobile device. Although the nurses highlighted that these issues provided significant barriers to long-term adoption of the TM system because of wasted time, they also expressed that there were timesaving aspects of the TM system.

#### Perceived Benefits by Nurses

When asked if the TM system could save them time in any regard, both nurses responded affirmatively:

Yes, absolutely [the system saved me time]: they did what [I] would have been asked to do, because you can see [the measurements].

This referred to the fact that in absence of the TM system, the nurse would have to take the patient’s physiological measurements, including blood pressure, heart rate, and weight, during their visit. With the TM system, the nurses had their patients’ recent and historical daily measurements available for viewing before and during the visit.

The 2 nurses stated that their home visits were enhanced because the TM system provided access to more patient data and information than they would have otherwise have had. The nurses noted that they “had knowledge of what [the patients] were doing in the past 2 weeks—so the trends were helpful”. The nurses attributed their ability to focus more on on-site education during the home visit to having this information readily available to them. One nurse explained:

[Each home visit] wasn’t about the numbers, because we were up to date with what the vitals were like and we went in knowing what the readings were...It was more about healthy lifestyle teachings and that kind of thing.

In this way, the nurses could focus their efforts during the home visit on helping the patients improve patient self-care and finding opportunities for *teachable moments*. In a particular encounter, the nurse described being able to work with the patient on issues surrounding diet choice and symptoms. The nurse also took an opportunity to introduce compression stockings to address what concerned the patient (ie, edema of their legs):

The year before, [the doctor] has been changing medication for [the patient] and he wasn’t feeling well. But during the study he was feeling well and his biggest complaint was the edema of his legs. He started reading all the labels on everything and reduced salt content and got some compression stockings and [was] willing to do whatever it took.

#### Willingness to Use the Telemonitoring System Long Term

Although nurses liked having the information provided to them through the TM system and saw the value add to their clinical work, the amount of additional work resulting from alerts triggered by the TM system and the follow-up communication that was required were a hindrance to their desire to continue using the system. One nurse described she liked the TM because she liked:

...having access to the information right on [her] Blackberry. It was nice to have the two-week trends to see what was going on.

When the nurses were asked if they would continue using the TM system if they were given a choice, one nurse answered:

If I had all “good” clients like the one I had, then yes! But if I had clients with the alerts every day, then no...You always end up regardless of if it’s part of the study or a regular client, you always have one with issues like this. This would be a definite determinant for me if I had a client like this I would be afraid of spending hours that you’re not being reimbursed to track down and leave messages, you are trying to do your due diligence but it’s beyond concerning and very stressful when the pressure is way up and you can’t get a hold of [the client] and that kind of thing.

The other nurse stated that she would be limited to use TM with a couple of clients at any 1 time because of the time commitment involved with TM. She stated:

...having the information, knowing how [the clients] were doing and if things were not right—but if you got an alert every day, it became a chore...But it’s nice having the information and I’d be willing to go with that again. But I wouldn’t want to have 10 patients like this. And getting alerts on the phone all the time. If I had a couple that would be fine, but if I had more, I would get frustrated because it really affects your day, because I have a ton of people I have to see and they’re waiting for you and if you’re on the side of the road trying to contact them, it is a bit difficult.

### Perceptions by the Patients

#### Patient Perceived Benefits and Continued Use of Telemonitoring

Patients stated that the use of the TM system resulted in them feeling more self-aware and confident in being able to manage their condition. It also helped them increase their interest in their health and their efforts to exercise. Their overall perception of the TM system was that it was easy to use, and they expressed that they enjoyed the added interaction with the nurses:

I found it very easy to check the [blood pressure] history. In fact, it was quite easy to track history.

They also indicated that they would keep using it if the TM system was available outside of this study. One participant responded to the question on whether or not they would want to keep using the TM system by saying, “Yes, I would, I found it very informative and it kept me on track to what my pressures and weights were, and at particular day or time.”

#### Patient Frustration With Alerts

Patients also commented on areas requiring improvement. Some technical issues led to poor perception of the TM system by some patients. Some patients stated that the system generated too many phone calls, and consequently, too many voicemails were left on their home phone as a result of nurses following up on the generated alerts. In other situations, the performance of the algorithm and the associated hardware was suboptimal and triggered too many false alerts. One patient stated:

That was a little frustrating...My morning BP was generally high...The very first time I’d get a call—I didn’t realize they had called and we’d gone out. I got back and I had over a matter of a couple of hours 14 messages, going to the Emergency immediately because my [blood] pressure was in danger.

The issue of numerous phone calls, alerts, and voicemails were particularly an issue for patients with full-time employment as they would be unavailable for a call from the nurses until the evening or the following day.

#### Feasibility of Data Collection and Analysis for Future Effectiveness Trial

All study data that were intended for collection to answer research question 2 were successfully collected, including the data from the patient chart reviews and values for the HOBIC, SCHFI, and MLFHQ. Of 6 patients, only 3 had complete HOBIC scores, and only 2 patients had complete pre-SCHFI and post-SCHFI and MLHFQ scores because of 3 patients dropping out of the study.

## Discussion

### Overview

This study’s main objective was to determine the feasibility of implementing a mobile phone–based TM system within a home care nursing agency for HF management. The interviews of home care nurses and patients, as well as other data sources collected for this study, provided insights into the factors that are associated with the feasibility of TM system implementation. Although there were some indications of perceived benefits of the TM system, such as nurses having access to additional patient data, the barriers outweighed the perceived benefits, resulting in only 6 patients being enrolled and half of them dropping out of the study, as well as frustration experienced by the home care nurses and patients.

### Research Question 1: Feasibility of Implementing a Mobile Phone–Based Telemonitoring System Within a Home Care Nursing Agency

As indicated in the TAM [21], external variables influenced the perceived usefulness and perceived ease of use of the TM system, which, in turn, determined the actual use of the TM system. Although both nurses and patients acknowledged the potential usefulness of TM to streamline and improve clinical management and found the TM system technology itself easy to use, this was outweighed by the 4 main external barriers that existed in this study. (1) There was a lack of a strong communication channel between the home care nurses and the patients’ physician. (2) Patients’ busy family and work situations and technical TM system issues led to challenges in patient retention. (3) Lack of interest and engagement by physicians led to patient recruitment challenges. (4) The home care agency had complicated workflows, which led to challenges in implementing the TM system. These barriers are largely dependent on the service delivery model that is used to provide the TM service (ie, home care nursing agency vs HF specialty clinic). Each of these barriers is discussed separately below.

#### Communication Challenges Between Nurses and Physicians

The difficulties that the home care nurses experienced in trying to contact the most responsible physician (MRP) led to frustration and backlogged the nurses. The MRP was required to set the initial target ranges for vital signs and verify modifications of the target ranges for each patient (inappropriate ranges resulted in false alerts). Home care nurses were not mandated to change a patient’s clinical care plan and, therefore, had to contact the patient’s MRP whenever the care plan had to be modified. There were also concerns that medical issues were not being addressed in a timely fashion, and the nurses would have to resort to advising the patients to visit the emergency department. Similar findings on the importance of effective nurse-physician communication to sustaining a TM program with home care nursing have been reported by Radhakrishnan et al [28].

To address these communication issues in future implementations, MRPs must be incentivized to participate, and a communication link between patients, nurses, and the MRPs must be ensured. Recent studies in health research literature have emphasized that when technology for delivering interprofessional communication is implemented without the necessary institutional guidance and support, they can just become a nuisance [29,30]. Furthermore, contingency plans should be put in place in cases where the nurses cannot reach the physician.

#### Patient Retention Challenges

Patient retention became a challenge for the study with 3 of 6 participants dropping out. The factors that appeared to influence patient attrition were employment status, age, having dependents, size of household, and early negative experiences with the TM system. All 3 of the participants who dropped out were relatively young (aged 35-58 years) and lived with members of their family who were disturbed by the TM phone calls. Of the 3 patients, 2 were also working full time. Therefore, the patients who dropped out may have felt *too busy* to properly participate in the TM program, or that the TM program was too disruptive to their daily lives, as also discussed by Sanders et al [31]. In addition, the 3 patients who dropped out experienced TM system issues at the beginning of their enrollment (eg, false critical 911 alerts, issues returning phone messages, or prior negative experiences with home care nursing). A study in 2012 corroborates that a major predictor of attrition in users of a TM system was their experience with it within the first 30 days of use [32]. In comparison, the patient group who did not drop out were all relatively older (aged 57-86 years) and experienced no issues related to TM or home care early on; 2 of 3 patients were also retired or not working and lived in a household of 2 or less.

All the study participants were followed by a cardiologist (referring physician to the study), were not already receiving home care, and were NYHA class II or III (none were class IV). This may have indicated that their HF management was already sufficient. The TM system may be of most benefit to patients who are not well managed and who have the most severe cases of HF. Future implementations should consider both demographic and health management variables when choosing the target population.

#### Recruitment Challenges

The project faced significant challenges in recruitment that led to the low enrollment rate of only 6 patients over 6 months. The recruitment process did not include the appropriate health care organizations or partners to facilitate quick recruitment of patients. The original intent to partner with the local community care access center to funnel eligible patients into the trial would have likely resulted in higher enrollment rates. In addition, there was little response from the local family physicians even after follow-up. Primary care physicians and cardiologists were unwilling to participate because of no tangible incentives, such as financial incentives, and the perceived increased workload. Successful physician recruitment was only achieved when it was direct and personalized; in this case, being directly referred by a colleague or clinical champion of the TM system. These barriers to physician adoption have also been identified in a previous study [33].

For a successful future implementation of similar innovations in home care nursing settings, a clinical champion should be identified, clinicians should be incentivized to participate, and a recruitment strategy must be put in place to streamline enrollment of patients, as described by Luxton et al [34].

#### Challenges Because of Complicated Workflows

Detailed workflow maps for the home care nurses were developed, which revealed several complexities to the study. For example, patients could have different nurses managing their care, and it was deemed not possible to assign specific nurses to the patients in the study. Therefore, it was necessary to train all the home care agency’s nurses and staff (>50) on the TM system and provide additional information to them for HF assessment. As another example, to bill the study for a phone call or home visit, approval from the general nursing supervisor was necessary, which delayed patient care. Most of these workflow issues could be addressed through the implementation of a dedicated TM team at the home care agency.

#### Perceived Benefits of Telemonitoring

Although the implementation barriers outweighed the perceived benefits and thus led to the low recruitment rate and high dropout rate, it should also be noted that both nurses and patients stated perceived benefits from TM, including improved patient self-awareness and confidence. In addition, the presence of the TM system changed the focus of the home visits from gathering symptoms and physiological measurements to more time spent on teaching patients how to perform appropriate self-care. Over the course of this study, nurses were able to better educate patients on how to self-manage their condition, such as managing their diet and exercise, and use of compression socks, which is a key component of patient empowerment and improved care [35].

The nurses also believed that the TM system provided a greater awareness of their patients’ health status, information to help decide on when a visit to the patient’s home was necessary, and trending information that they could discuss with the patients. These results are in alignment with what other researchers have identified when using TM for patients with diabetes [36]. For half of the home visits, the nurses thought that by using the TM system, telephone calls could have replaced physical home visits, which could lead to potential financial savings. However, other studies have shown that although telephone follow-up can result in patient empowerment, they do not necessarily reduce readmissions [37].

#### Implications of Service Delivery Models

The implementation barriers described above of deploying the TM system in a home care nursing setting were in contrast to the relatively seamless deployment of the same TM system in a large specialty HF clinic using the same technology [38,39]. This was mainly because of the clinical buy-in, clinical mandate (ie, reduction of rehospitalization), advanced disease severity, and the infrastructure of the specialty clinic, including salaried nurse practitioners. During a randomized controlled trial (RCT) in the specialty clinic, 100 HF patients were recruited in 6 months compared with the 6 patients recruited in 6 months in the home care nursing setting. Of the patients in the intervention group of the RCT, only 3 of 50 patients dropped out of the program compared with 3 of 6 in the home care nursing setting. The site preparation was also minimal in the specialty clinic because of the clinical buy-in and single site, compared with the enormous effort of integrating with the clinical workflows and training the nonspecialized nurses in the home care nursing setting. It is evident that the type of service delivery model plays an important role in the success of a particular innovation.

### Research Question 2: Feasibility of a Future Effectiveness Trial

The second intent of this feasibility trial was to inform whether the project should proceed to the next phase of an effectiveness trial. Therefore, it was important to first determine how feasible it would be to collect and analyze the required data for an effectiveness trial [40]. This study found that the data that would be necessary for such an effectiveness trial, including the questionnaire data and chart review data, could be collected and analyzed successfully. The nurses were also willing to complete the data forms after each home visit and telephone call to the patient, providing insights into how TM could be implemented by a home care agency. The numerous feasibility challenges of implementing HF TM into home care nursing discussed above, including recruitment, physician buy-in and communication, workflow integration, and retention, must be addressed before an effectiveness trial.

### Limitations

Although much was learned in terms of the feasibility of implementing a TM system, a larger number of patient and nurse participants would have provided further insights into their perceptions of TM. In addition, the same cardiologist referred all patients who participated in this study. However, the barriers experienced in recruiting patients and obtaining buy-in from physicians in this model was an important finding in terms of feasibility. Another limitation is that the deployment involved a single home care agency and some of the workflow issues experienced may be specific to that agency.

### Conclusions

Although the study revealed examples of the perceived benefits of the TM system to improve care by home care nurses, the many implementation barriers encountered outweighed the perceived benefits. These barriers must be resolved to successfully implement TM in the home care agency. The main lessons learned from this study included the necessity to have physician buy-in, as well as streamlined processes to recruit and manage patients. Although enormous effort was spent to recruit patients for the study, this was largely unsuccessful. To promote physician buy-in, incentives to participate must be developed, which would also mitigate the nurse-physician communication issues that existed in the trial. The demographics of the patients should be considered when deploying such a program to help ensure adherence and reduce dropouts. The establishment of a core group of TM nurses would help address the complicated workflow issues identified. The outcomes from this trial and a previous trial in an HF specialty clinic using the same intervention emphasize the importance of feasibility trials when deploying in different service delivery models to identify context-specific barriers.

## Abbreviations

HF: heart failure
HOBIC: Health Outcomes for Better Information and Care
MLHFQ: Minnesota Living with Heart Failure Questionnaire
MRP: most responsible physician
NYHA: New York Heart Association
RCT: randomized controlled trial
SCHFI: Self-Care of Heart Failure Index
TAM: technology acceptance model
TM: telemonitoring
